# Successful Bilateral Sperm Retrieval in a Hypogonadal Patient with Non-Obstructive Azoospermia Showing Normal Serum 17-Hydroxyprogesterone Levels Suggestive of Normal Intratesticular Testosterone Production: A Case Report

**DOI:** 10.3390/jcm12103594

**Published:** 2023-05-22

**Authors:** Ettore Caroppo, Giovanni M. Colpi

**Affiliations:** 1 Asl Bari, Reproductive Unit, Andrology Outpatients Clinic, PTA “F Jaia”, 70014 Conversano, Italy; 2 Next Fertility Procrea, Andrology and IVF Center Unit, 86900 Lugano, Switzerland; gmcolpi@yahoo.com

**Keywords:** non-obstructive azoospermia, hypogonadism, 17 OHP, intratesticular testosterone, sperm retrieval, case report

## Abstract

The impact of hypogonadism on the probability of retrieving testicular sperm from patients with non-obstructive azoospermia (NOA) is still a matter of debate. Conflicting evidence in this field may be justified by the striking differences between serum and intratesticular testosterone (ITT) levels found in men with severe spermatogenic dysfunction, so that normal ITT levels may coexist with low serum testosterone levels. Here we report the case of a patient with NOA with a steadily reduced serum testosterone level irresponsive to hormonal stimulation with human chorionic gonadotropin. Supported by his normal serum 17-hydroxyprogesterone (17 OHP) levels, previously suggested to be marker of ITT levels, microdissection testicular sperm extraction was performed for both testes on two separate occasions, resulting in the retrieval of enough sperm for ICSI. Three ICSI cycles were then performed, one blastocyst was transferred, and five were cryopreserved. This case report suggests that normal serum 17 OHP levels, being suggestive of normal ITT levels, may support the decision to proceed with surgical sperm retrieval in hypogonadal patients with NOA, even for those irresponsive to hormonal treatment.

## 1. Introduction

Azoospermia due to severe spermatogenic dysfunction, also termed non-obstructive azoospermia (NOA), is a challenging clinical condition for which no medical treatment has been proven effective. The surgical retrieval of testicular sperm, and their subsequent use for intracytoplasmic sperm injection (ICSI), represents the only available strategy to enable these patients to father their own biological children. However, due to the anatomical singularity of the testicular parenchyma in these cases, characterized by the (eventual) occurrence of sporadic areas of residual spermatogenesis dispersed among thin and atrophic seminiferous tubules (STs), finding sperm is not always easy, even when the most effective surgical technique, namely microdissection testicular sperm extraction (mTESE), is used. 

Although no pre-surgical marker can reliably predict the outcome of mTESE [1,2], the possible impact of hypogonadism on the probability of successful sperm retrieval is still a matter of debate. Reifsneyder et al. evaluated 736 patients with NOA undergoing mTESE, 348 of them with hypogonadism (baseline testosterone (T) level lower than 300 ng/dL). Of these, 307 hypogonadic patients received hormonal treatment and 252 (82%) responded with a serum T of at least 250 ng/dL before mTESE, while 55 did not respond: sperm retrieval rate was, however, comparable in both subgroups [3]. Similar results were recorded by Althakafi et al., who evaluated 421 patients, including 181 with hypogonadism, but did not find a difference in SRR between those with normal and low T levels (SRR 38.6% vs. 40.3%, *p* = 0.718) [4]. On the other hand, Mehmood et al. and Çayan et al., evaluating 264 and 327 patients respectively, found that SRR was significantly lower in men with low baseline T levels compared with those who had normal baseline T levels (40.6 vs. 57.25, *p* = 0.0068, and 40.5% vs. 65.9%, *p* < 0.0001, respectively) [5,6]. 

These seemingly conflicting results may, however, be explained by hypothesizing that some patients with subnormal serum testosterone levels may nevertheless have normal intratesticular T (ITT) levels that may be sufficient to support spermatogenesis. Indeed, it was found that serum and intratesticular T levels are not correlated in men with NOA, with ITT being 94 fold higher than serum T [7]. A previous study found that men with severe spermatogenic dysfunction, such as patients with Klinefelter syndrome (KS) or Sertoli cell-only syndrome (SCO) had higher ITT levels than men with normal spermatogenesis, while their serum T levels were significantly lower compared with those of normal men [8].

Obtaining the ITT levels from hypogonadal patients with NOA could, therefore, be advisable to improve their management. ITT levels are usually obtained by performing testicular fine needle aspiration or by using testicular biopsy specimens, with the inherent limitation of the invasiveness of the technique and the risk of testicular damage in the former strategy, and of ITT levels being available only after surgery for the latter. Recently, however, measurement of the circulating levels of 17-hydroxyprogesterone (17OHP), a T precursor, has been proposed as an indirect biomarker of ITT levels, given that 17 OHP is likely to be of mainly (70%) testicular rather than adrenal origin in men. Interestingly, serum 17 OHP levels were found to be undetectable in men receiving exogenous testosterone replacement therapy [9] and to increase after clomiphene citrate (CC) or human chorionic gonadotropin (hCG) treatment in hypogonadal men [9,10].

Here we report the case of a NOA patient with hypogonadism resistant to hormonal treatment, whose normal 17 OHP serum levels, suggestive of normal ITT levels, prompted us to proceed with mTESE despite very low serum T levels. MTESE was performed on both testes on two separate occasions, with the successful recovery of sperm and their further use for ICSI cycles. 

## 2. Case Report

A 32-year-old male patient was referred to our center for male factor infertility. He had received a diagnosis of non-obstructive azoospermia and primary hypogonadism in 2013, due to low serum T levels (159 ng/dL), high FSH (52.9 mIU/mL) and LH (40.3 mIU/mL) serum levels, and lack of sperm in the ejaculate on two separate occasions, with semen analysis showing normal semen volume (3.8 mL) and pH (7.8). Genetic screening tests did not reveal chromosomal abnormalities, Y chromosome microdeletion, nor CFTR mutations. Despite the low T serum levels, the patient reported normal sexual libido and erectile function. However, following his endocrinologist’s advice, he started a treatment with long-acting testosterone undecanoate (Nebid 1 gr—Bayer) 1 ampoule every 12 weeks, with resulting normal serum T levels (382 ng/dL) during treatment. However, the patient discontinued treatment three years later due to side effects (hot flushes) and, despite consistently low serum T levels over the following three years, did not start any further treatment.

In February 2021 he came to our attention, together with his 28-year-old female partner, seeking infertility treatment. He reported normal sexual activity and no symptoms of hypogonadism. The physical examination revealed normal secondary sexual characteristics, small testes (2 mL), body mass index 24.6 kg/m^2^. Since serum T was 128 ng/dL, he received hCG (Gonasi, Ibsa) 2000 I.U. two times a week (CC treatment was not feasible due to hypercholesterolemia). His serum 17 OHP was, anyway, normal (88 ng/dL). Serum 17OH-P was measured by ELISA: the intra- and inter-assay coefficients of variation were 4.7% and 9% respectively. The assay range provided by the manufacturer was 5–160 ng/dL. HCG treatment, however, was unable to restore the serum T levels to the normal range, even when the dosage was raised to 2000 I.U. three times a week and to 5000 I.U. every fifth day; nevertheless, serum 17 OHP remained steadily in the normal range (data are displayed in Table 1). 

**Figure 1:**
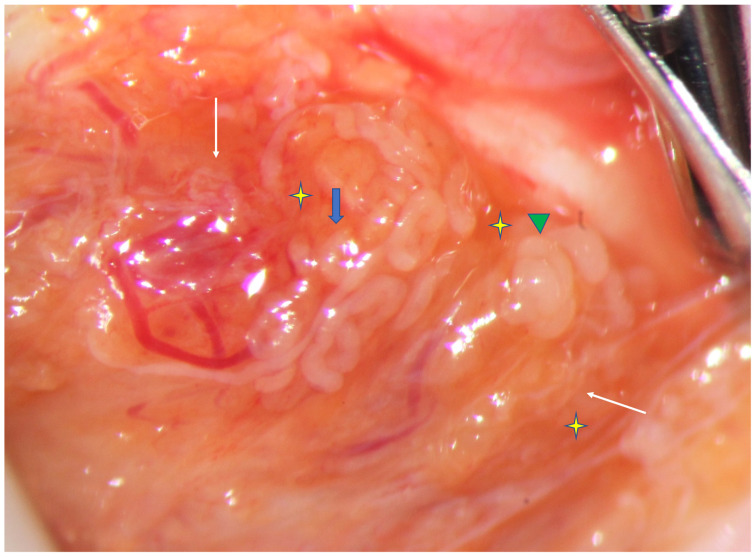
Testicular parenchyma at high magnification (×36) during mTESE: rare dilated STs (*green triangle*) and some with smaller diameter (*blue arrow*) scattered in a parenchyma prevalently composed of thinner or atrophic tubules (*white arrows*). The orange color of the testicular tissue is suggestive of a high concentration of Leydig cells. The dilated and slightly dilated STs are located close to the vessels and to Leydig cells (*yellow stars*).

Since serum 17 OHP was normal, we proceeded with mTESE on the right testis. Viewed at high magnification (36×), the testicular parenchyma was mainly composed of atrophic, thin seminiferous tubules (STs), surrounded by a high number of Leydig cells, as suggested by the orange color of the parenchyma (Figure 1). Deeper exploration of the parenchyma allowed the identification of small areas containing dilated STs, mostly located close to aggregates of Leydig cells. Testis histology performed on the STs representative of the overall appearance of the testicular parenchyma showed early maturation arrest. ICSI was performed on the same day with fresh testicular sperm, while surplus sperm were cryopreserved (three paillettes). The presence of motile sperm was checked: if no motile sperm were found, sperm were processed with theophylline. If no motile sperm were obtained after this processing, the hypo-osmotic swelling test was used to select viable sperm. Sperm with normal morphology were preferentially used for ICSI, when available. In total, 13 metaphase II (MII) oocytes were injected out of the 14 oocytes retrieved (1 oocyte was at the MI stage), 2 were fertilized, but no embryo was available for transfer. A further ICSI attempt was performed with cryopreserved testicular sperm following adjustments in the ovarian stimulation protocol: 14 oocytes were retrieved, 11 were MII, 5 out of 11 were fertilized, and 2 blastocysts 4AA were available. The first blastocyst was transferred, but no pregnancy was achieved.

Before proceeding with the transfer of the residual blastocyst, the patient asked for a second mTESE attempt on the contralateral testis, to store sperm for further ICSI attempts before starting testosterone treatment. MTESE was therefore performed on the left testis, whose STs picture was comparable to that of the right testis, and sperm were successfully retrieved. ICSI was performed on 13 MII oocytes (out of 16 total oocytes retrieved), 9 of which were fertilized, and 4 blastocysts 4AA were cryopreserved.

## 3. Discussion

The present case report suggests that residual spermatogenesis in patients with NOA may be sustained by normal intratesticular T levels even when serum T levels are very low. We did not directly measure the ITT levels, but relied upon the serum 17 OHP level assay as marker of ITT levels, according to the evidence arising from previous studies [9,10]. According to the results of a cross-sectional analysis of 247 fertile controls, who determined the 25th, 50th, and 75th percentile for serum 17-OHP (34.2, 55.72, and 105.5 ng/dL respectively) [11], our patient had serum 17 OHP levels always comparable to those of fertile controls (Table 1). 

Testosterone is required for spermatogenesis to proceed beyond meiosis. In men with NOA, however, serum T levels may be reduced as direct consequence of severe spermatogenic dysfunction and the associated Sertoli cell dysfunction, which disrupts the balance between testicular inhibin and activin in favor of the latter at a paracrine level: the enhanced activin signaling determines a decrease in cytochrome P450 17 alpha-hydroxylase and 17, 20 lyase activity, with consequent reduced T secretion [12]. Such a mechanism, although resulting in a reduction of serum T levels, may not affect spermatogenesis if ITT concentrations are preserved. Indeed, ITT levels in men with NOA are significantly higher than T concentration in the peripheral circulation. In addition, even very low ITT levels have been found to support spermatogenesis in mice, while total blockage of T activity through administration of the antiandrogen flutamide halted spermatogenesis at the round spermatid stage [13].

Our patient had patchy spermatogenesis in both testes, and when his testicular sperm were used for ICSI, they were able to fertilize eggs and to promote the development of embryos to the blastocyst stage. As demonstrated by the improved ICSI outcome in the second and third ICSI cycle, the fertilization failure occurring in the first ICSI cycle could have been due to female factors: further adjustment in patient management and in ovarian stimulation protocols led to a progressive increase in fertilization rates (from 15% to 45% and finally to 69%) as well as in blastulation rates (from 0% to 44%). At present, the couple have not proceeded further with the transfer of the residual blastocysts; however, the number of blastocysts obtained represents a favorable outcome. It has demonstrated that when at least a blastocyst is obtained in these patients, the euploidy rate is not affected by the severity of the male factor infertility, and pregnancy and live birth rates per transfer are comparable to those of infertile men with less severe forms of spermatogenic dysfunction [14]. 

## 4. Conclusions

To the best of our knowledge, this is the first study documenting successful bilateral sperm retrieval in a patient with NOA with frank hypogonadism irresponsive to hormonal treatment, but with normal serum 17 OHP levels suggestive of normal ITT levels. The results of this case report suggest that surgical sperm retrieval may be attempted when serum T levels are low but serum 17 OHP levels are normal. Further studies in a large cohort of patients with NOA are required to verify the generalizability of the present results.

## Figures and Tables

**Table 1:** Patient’s hormonal parameters over time and following different therapeutical strategies.

Date	FSH (mIU/mL)	LH (mIU/mL)	T (ng/dL)	17 OHP (ng/dL)	E2 (pg/mL)	Treatment
May 2013	52.9	40.3	159	N.A.	N.A.	
October 2014	N.A.	N.A.	382	N.A.	N.A.	Long-acting testosterone undecanoate
July 2019	48.5	41.3	125	/	N.A.	None
Feb 2021	46.14	27.56	138	88	21	None
April 2021	49.46	29.52	142	74		hCG 2000 IU b.i.w.
June 2021	45.03	22.47	84	92	<20	hCG 2000 IU t.i.w.
August 2021	N.A.	N.A.	136	N.A.	N.A.	hCG 5000 IU every 5th day
January 2022	N.A.	N.A.	98	N.A.	N.A.	hCG 5000 IU every 5th day
October 2022	36.75	19.42	56	66	N.A.	hCG 5000 IU every 5th day

N.A. not available.

## Data Availability

Not applicable.
